# Fads in Medicine

**Published:** 1915-11

**Authors:** Henri Bogart

**Affiliations:** Shelbyville, Ill.


### EDITORIAL DEPARTMENT.

MRS. F. E. DANIEL, Publisher and Managing Editor.
DR. R. H. L. BIBB, Associate Editor.

CONTRIBUTING EDITORS:

Dr. H. Sheridan Baketel, New York, editor *Medical Times;* Dr. M. H. Boerner, Austin, Texas; Dr. G. Henri Bogart, Paris, Ill.; Dr. M. M. Carrick, Dallas, Texas; Dr. Edward H. Carey, Dean Medical Department, Baylor University, Dallas, Texas; Dr. W. L. Crosthwaite, Waco, Texas; Dr. T. D. Crothers, Hartford, Conn.; Dr. H. W. Cummings, Hearne, Texas; Dr. Oscar Dowling, New Orleans, President Louisiana State Board of Health; Havelock Ellis, M. D., London, Eng.; Dr. May Agnes Hopkins, Dallas, Texas; Dr. A. W. Fly, Galveston, Texas; Dr. G. B. Foscue, Waco, Texas; Dr. E. S. Goodhue, Holualoa, Kona, Hawaii; Dr. M. B. Grace, Seguin, Texas; Dr. Joe Gilbert, Austin, Texas; Dr. Charles H. Hughes, St. Louis, editor *Alienist and Neurologist*; Dr. James G. Kiernan, Chicago; Dr. George H. Lee, Galveston, Texas; Dr. John T. Moore, Houston, Texas; Dr. Frank Paschal, San Antonio, Texas; Dr. John Preston, Austin, Texas, Superintendent State Lunatic Asylum; Dr. G. Wilse Robinson, Kansas City, Mo.; Dr. Bacon Saunders, Fort Worth, Texas; Dr. Allen J. Smith, Philadelphia, Medical Department, University of Pennsylvania; Dr. Z. T. Scott, Austin, Texas; Dr. Ralph Steiner, Austin, Texas, President Texas State Board of Health; Dr. Walter S. Sutton, Kansas City, Mo.; Dr. A. E. Sweatland, Nacogdoches, Texas; Dr. John S. Turner, Dallas, Texas; Dr. Charles Venable, San Antonio, Texas.

---

The value of medical organizations for every physician of reputable standing is clear. Their leaders are men foremost in the profession. Their journals publish promptly records of the latest investigations. Their meetings quicken and foster and encourage initiative and a response to the ideals of the profession. While medical societies have existed for centuries in one form or another, their rapid development within twenty years is significant of the increasing appreciation of physicians in the personal and professional advantages to be gained from organization.

It could hardly be otherwise in an era of unions and organizations. From the newsboys to the churches a "get together" movement is in vogue. Each separate activity, commercial or altruistic, for its own advancement realizes that union implies power.

Each association, club, union, society, or order, in purpose, methods, and spirit reflects the ideals, achievements and attitude of its composite membership. Medical societies, noblesse oblige, rank in ideals among the highest and noblest of all, if, indeed, they do not lead. The physician from the nature of the service rendered has intimate knowledge of human needs and human weaknesses. In the present stage of medical science he sees more clearly than anyone else the remedy not only for the individual, but for society as a whole. Medical organizations show this grasp of social conditions and their activities forecast social changes. In addition, these organizations do the practical thing. They study medical problems of the section represented, whether it be a State, a country, or a specially selected unit of territory. They publish in acceptable and authoritative form the results of proved investigations. They foster an esprit de corps among the members which otherwise could not exist. They give opportunity for professional condemnation of unethical practices and public health evils. They increase the confidence of the trustworthy and put fear in the heart of the quacks and the dishonest. They lift to a higher plane of thought and action those who participate in their activities.

From the day even of Aesculapius, the healing art to the average man has been thought of as mysterious. Through the ages this idea was fostered by conditions. The medical man, though wise to "signs" and often with insight and the healing gift, as to cause in many instances, knew no more than his neighbor of the tradesman's guild. But now, because of the courage, self-sacrifice, and toil of the physician himself, we know enough to teach clearly and widely the causes of many human ills. The medical society offers opportunity for the dissemination of this kind of knowledge; it affords a medium of communication between the average man, unlearned in medical science, and the physician-scientist who would teach the simple and practical truths of hygiene and disease prevention.

Conferences of doctors make known to the public the broader aims of the physician and his interest in community welfare, and wider still, in society. When an association, county, State, or national, condemns a practice which is inimical to the health of the persons duped, the condemnation means both public enlightenment and public protection. The spring records of many county and State medical associations throughout the South and other sections show that there is an awakening as to the evil of the patent nostrum, so-called medicine. Resolutions commending the general movement against these frauds have been unanimously adopted and publicity given to this expression of professional disapproval. It is significant of a persistent and organized campaign which will continue until the quack and the nostrum are no more.

Though medical organizations have grown in number, in strength of purpose, and in power, especially during the past decade, the membership is not so large as it should be. It is estimated only about one-fourth of the physicians of the country are active members of their respective societies. There are some good reasons, one being the difficulty which a doctor experiences in attending meetings. But where so much is to be gained for the physician himself, for those he serves, for the community and society, no trivial circumstance should prevent affiliation with the local, State and national associations. Happily, the organizations are proving themselves as to their value, and the increasing membership is gratifying to all who are interested in the advancement of the profession and the health of our citizens.—*Dr. Oscar Dowling.*

---

### Fads in Medicine.

BY DR. HENRI BOGART, SHELBYVILLE, ILL.

The medical profession is peculiarly likely to swing to radical extremes. The really learned medical man who is honest with himself, who has learned to commune with himself as with another knows the limitations of our science. What is rheumatism? Cancer? Is leprosy contagious? Or infectious? What is pellagra? How shortly since we learned the causation of yellow fever? And malaria? How old do you have to be to know the useless, cruelly burdensome quarantine and disinfection of yellow jack? Those men, some of them readers of this editorial, took part in these things. They were sternly earnest and honest in what they did. They rushed into the breach to battle with death and freely laid down their lives. They believed they knew just as we believe we know.

Doctor, delve deep into your soul and memory a bit. How often have you had cases that you did not diagnose fully and correctly, how often have you felt misgivings concerning your verdicts?

Dr. Stiles is not an old man, but how long was hookworm called malaria?

Medicine has made vaster strides than any other science of recent years. But subconsciously we realize our own impotence when we would be so strong, so sure. So, earnestly seeking to serve, to save, we clutch desperately at the promise of the new thing.

Antiseptic surgery led us to irrigate wounds of trauma or operate with bichloride 1-1000, even at times 1-500. We were like drowning men clutching at straws. We disinfected apartments, and clothing, and person to guard against tubercular bacilli, when the post-mortem showed that practically all at middle age have been infected, and the many have experienced spontaneous cure. We welcomed salvarsan as an absolute specific cure, when we now know that it is exceedingly useful to ameliorate, to aid other remedies.

Groping in the dark, we knew mercury, quinine, arsenic, sulphur, and iodine as alteratives, something that altered diseased conditions; now we know it was because these remedies are germicides that within certain narrow limits are fatal to disease cells without serious damage to living tissue cells. And in our deep desire to help, to cure, in partial realization of our narrow scope of absolute knowledge, we rush, stampede to that which gives promise of an enlarged horizon.

The tendency of the medical profession to leap forward to welcome new remedies and methods is the best promise of the future. The man who "knows it all," who has become self-centered, is past growth; while he who is ever seeking new and better treatment, remedies, and appliances is open to grow as the science grows; he has not become muscle bound.

So, while our beloved science of healing is advancing by leaps, we stumble, oftimes in our laudable anxiety to widen and strengthen our skill.

But, doctor, on the other hand, remember that there is no panacea; hold that as an axiom. So many of us, especially we who know the years that come and go, have seen so many fads of treatment pass into a forgotten limbo that we demand the test of time to winnow the precious grains of truth and real advance from the chaff of error ere we give unqualified indorsement. While some of us embrace each new promise, others stand with equal disbelief after a new thing is proven. The difference of attitude is merely temperamental. Calm, dispassionate reason would take the middle ground, and I am proud that our profession is sufficiently honest, down in its inner consciousness, to reconize its limitations and grasp at each new idea that is advanced.

